# Mechanical performance of 3D-printed and milled resins for pediatric provisional restorations

**DOI:** 10.1038/s41598-025-22841-2

**Published:** 2025-11-06

**Authors:** Ji-Su Song, Yooseok Shin, Jee-Hwan Kim, Ko Eun Lee, Jiho Lee, Jinhong Min, Hoon Kim, Je Seon Song

**Affiliations:** 1 Department of Pediatric Dentistry, Yonsei University College of Dentistry, 50-1 Yonsei-ro, Seoul, 03722 Republic of Korea; 2 Department of Conservative Dentistry, Yonsei University College of Dentistry, Seoul, Republic of Korea; 3 Department of Prosthodontics, Yonsei University College of Dentistry, Seoul, Republic of Korea; 4 Research Institute of Agriculture and Life Sciences, College of Agriculture & Life Sciences, Seoul National University, Seoul, 08826 Republic of Korea; 5 Department of Research Planning , Graphy Inc. R&D Center, Seoul, Republic of Korea

**Keywords:** 3D-printed crowns, Mechanical properties, Dental materials, Provisional dental materials, 3D printing, Pediatric dentistry, Materials science, Preclinical research

## Abstract

Given the growth-related challenges in pediatric and adolescent patients and the increasing adoption of digital workflows, durable yet adaptable materials are needed for provisional restorations. This study evaluated the mechanical performance of two 3D-printed resins, Graphy TC-80DP (GP) and NextDent C&B MFH (ND), and a milled nano-hybrid resin, Mazic Duro (MD). A multidimensional assessment was conducted, including fracture resistance, flexural strength, flexural modulus, viscoelasticity, and polymerization behavior. ND showed the highest fracture resistance, MD exhibited superior flexural strength and flexural modulus, and GP demonstrated faster polymerization and greater thermal stability. These findings suggest that both 3D-printed and milled resins are clinically viable for pediatric provisional restorations, with material selection depending on occlusal load, patient age, and functional demands.

## Introduction

The oral environment in pediatric and adolescent patients is constantly evolving due to the eruption of permanent teeth, craniofacial growth, and changes in occlusal relationships and bite force^1–3^. These developmental dynamics necessitate provisional restorative solutions that not only restore function and esthetics but also allow for flexibility in future treatment planning. Accordingly, long-term provisional restorations are considered more appropriate for growing patients^1,4,5^. In growing patients, provisional or full-coverage restorations are clinically important for maintaining function and esthetics and for protecting pulp-treated teeth until definitive care, as supported by contemporary pediatric restorative guidelines and recent crown reviews^6,7^.

Occlusal forces in children increase with age, reaching up to 300–500 N, and may exceed 400 N in the molar region^8^. Parafunctional habits such as bruxism and clenching introduce additional mechanical stress, underscoring the need for restorative materials with sufficient fracture resistance, flexural modulus, and viscoelastic behavior^9^. Materials with excessively high flexural modulus can concentrate stress and damage surrounding tissues, whereas insufficient flexural modulus may lead to deformation during mastication^3,10^.

Preformed metal crowns (PMCs) have been widely used as long-term provisional restorations due to their durability and ease of placement. However, their limitations—such as suboptimal marginal adaptation, poor esthetics, and limited adaptability to dynamic occlusion—raise concerns regarding their long-term use in growing patients^1,11–13^. CAD/CAM-milled nano-hybrid resins have emerged as an alternative, offering improved mechanical properties and esthetics. Nevertheless, this approach involves significant material waste and limited customization^10,14–17^. In recent years, 3D printing technology, particularly digital light processing (DLP)-based resins, has gained attention due to its capacity for efficient fabrication, reduced material consumption, and customized prosthesis production^18^.

3D-printed resins offer high fracture resistance, favorable flexural properties, viscoelasticity, and consistent photopolymerization behavior. Their ability to closely match patient-specific anatomy, combined with improved esthetics and biocompatibility, makes them a promising option for long-term provisional restorations in pediatric dentistry^19–25^. However, data on the clinical performance and mechanical reliability of 3D-printed resins remain limited. Most previous investigations have evaluated only isolated mechanical properties under static conditions, with few assessing dynamic or thermal responses. Moreover, inconsistent reporting of resin formulations and post-curing protocols makes it difficult to compare results across studies. Importantly, pediatric and adolescent applications are underrepresented, and comprehensive comparative analyses of 3D-printed and milled resins under clinically relevant mechanical and thermal conditions are lacking. While previous studies have compared fabrication methods or assessed basic mechanical traits, few have simultaneously analyzed both 3D-printed and milled materials under conditions that reflect pediatric clinical needs. In particular, limited data are available on the viscoelastic and thermal behavior of 3D-printed resins across dynamic intraoral conditions.

To address this gap, the present study compares the mechanical properties of two 3D-printed resins, Graphy TC-80DP (GP) and NextDent C&B MFH (ND), with a CAD/CAM-milled nano-hybrid resin, Mazic Duro (MD). Key parameters such as fracture resistance, flexural strength, and flexural modulus were assessed, alongside dynamic mechanical analysis (DMA) and UV rheology to evaluate viscoelastic behavior, thermal stability, and polymerization kinetics. We hypothesized that 3D-printed resins and CAD/CAM-milled resins would exhibit comparable mechanical properties under clinically relevant conditions. By testing this hypothesis, the study seeks to guide the selection of provisional restorative materials that address the unique biomechanical demands of pediatric and adolescent patients. This multidimensional evaluation further provides clinically relevant insights into their adaptability and mechanical durability under long-term functional conditions, supporting clinicians in material selection.

## Materials and methods

**Fig. 1:**
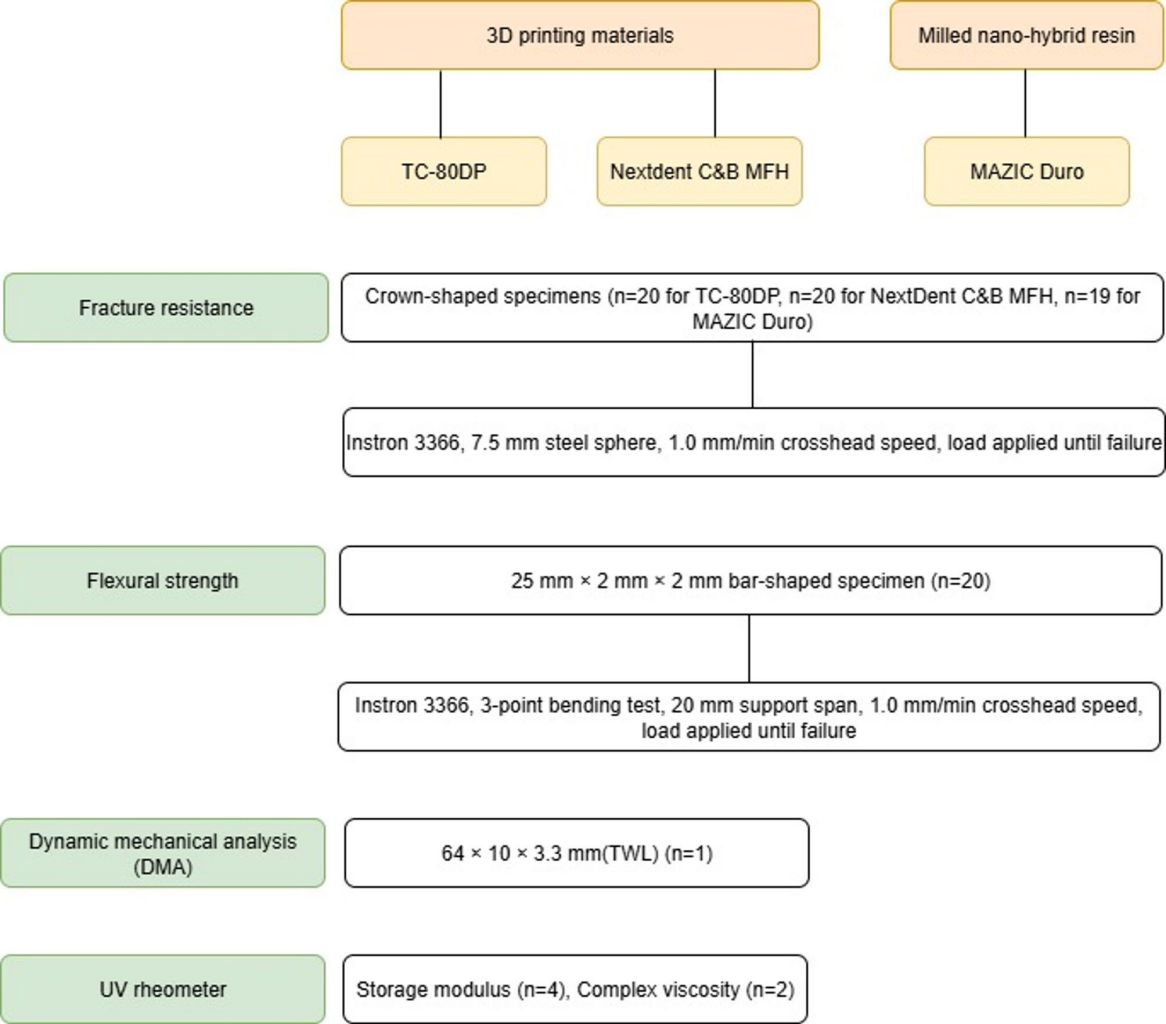
Experimental flow chart.

An experimental workflow is shown in Fig. 1. These assays were chosen to reflect dominant intraoral failure pathways in pediatric full-coverage restorations: (1) localized, high-magnitude occlusal contacts—exacerbated by bruxism—can trigger catastrophic cracking; therefore, a compressive load-to-fracture test was used. (2) Routine mastication subjects crowns to bending; thus, flexural strength and, particularly, flexural modulus were measured to capture resistance to deformation (too low favors bending/fatigue, whereas too high concentrates stress at the margins). (3) Because pediatric function involves time-, frequency-, and temperature-dependent loading, DMA across clinically relevant frequencies and temperatures quantified storage/loss moduli and tan δ. (4) Finally, UV rheology characterized polymerization kinetics and network development (storage modulus/complex viscosity), which underpin early-age handling, thermal stability, and long-term viscoelastic behavior.

### Materials

Three different resin materials for long-term provisional fixed restorations were selected. Among them, two were 3D-printed resins: Graphy TC-80DP (GP; Graphy, Seoul, South Korea) and NextDent C&B MFH (ND; Vertex-Dental B.V., Soesterberg, Netherlands). Additionally, a CAD/CAM-milled nano-hybrid ceramic resin, Mazic Duro (MD; Vericom, Gangwon-do, South Korea), was included. The detailed composition and manufacturers of the materials used in this study are presented in Table 1.

**Table 1:**
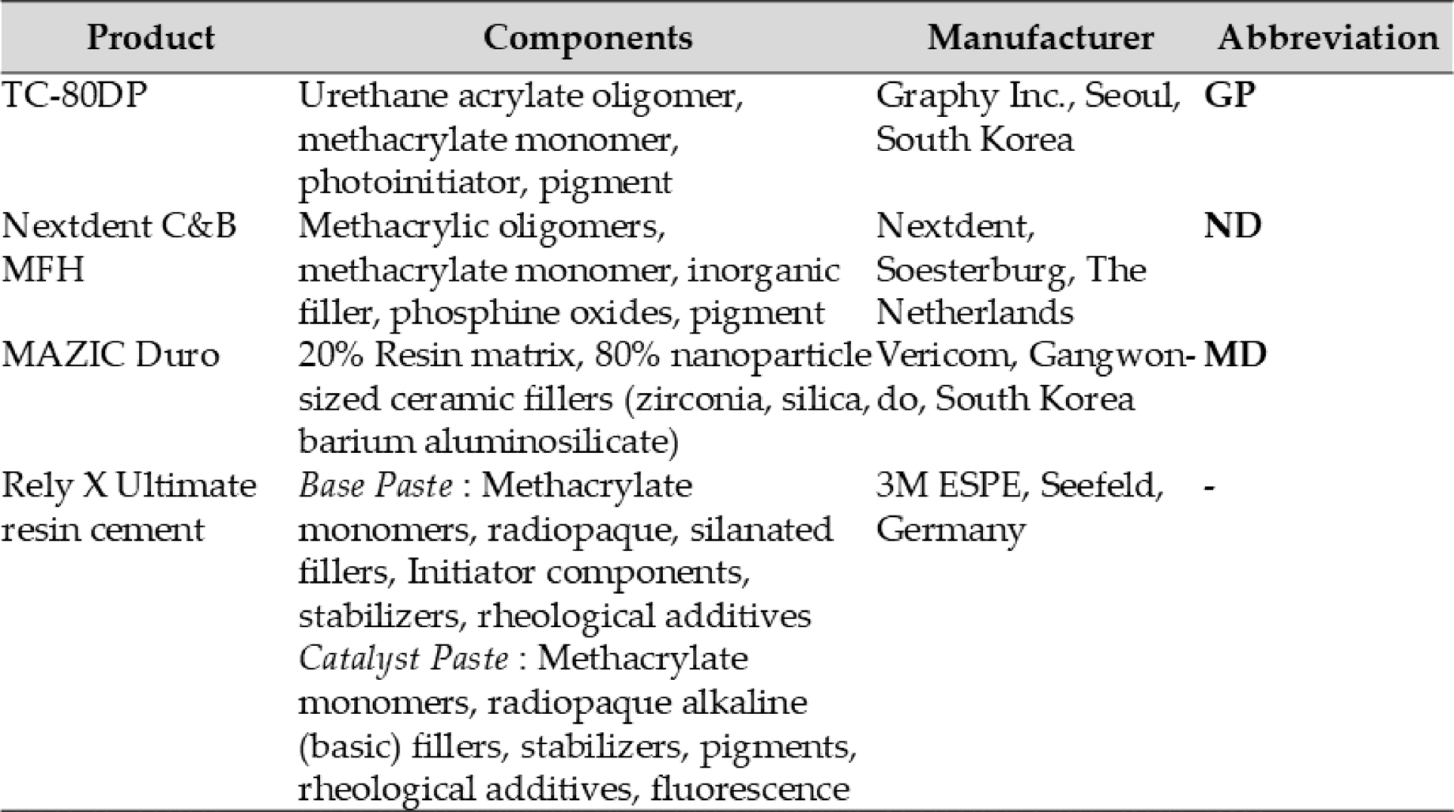
Components and manufacturers of materials used in thestudy.

### Sample preparation

**Fig. 2:**
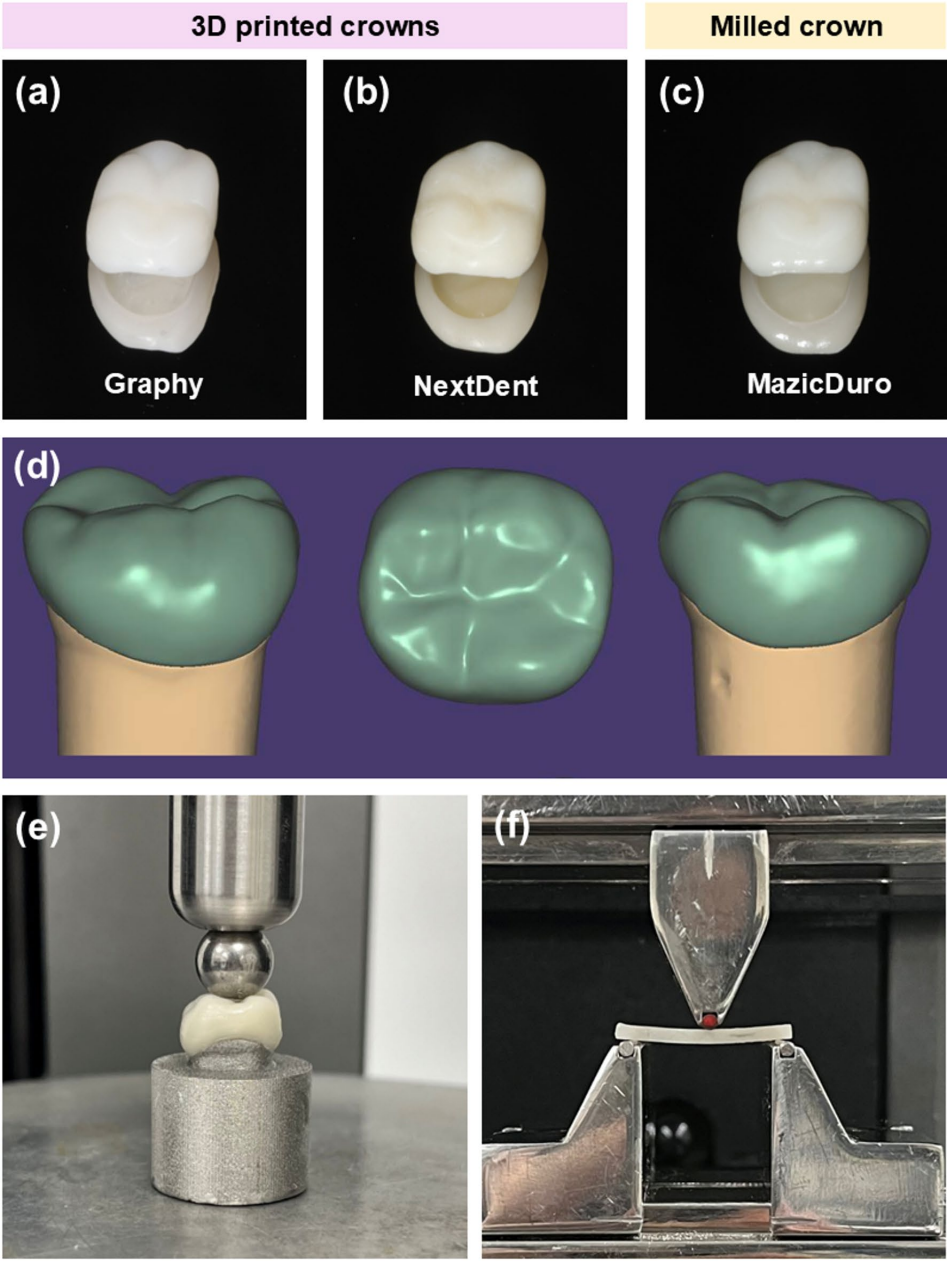
(**a**–**c**) Crown specimens fabricated from Graphy and NextDent (3D-printed) and Mazic Duro (milled); (**d**) CAD designs of crown and bar-shaped specimens for fracture and flexural testing; (**e**) fracture-resistance test using a stainless-steel ball; and (**f**) three-point bending test setup for flexural-strength evaluation.

For the fracture test, crown-shaped specimens were designed using Exocad software (Exocad GmbH, Darmstadt, Germany) and fabricated with an occlusal thickness of 1.5–2.0 mm and axial thickness of 1.5 mm^14^ (Fig. 2d). For the three-point bending test, bar-shaped specimens were designed using 3D modeling software (NBEE; Uniz, San Diego, CA, USA) following the ISO 10,477 standard (25 × 2 × 2 mm)^14,26–28^. Subsequently, using the generated STL files, the two 3D-printed resin materials, GP and ND (Graphy TC-80DP and NextDent C&B MFH, respectively), were fabricated using each manufacturer’s recommended digital light processing (DLP) 3D printers as shown in Table 2. The milled nano-hybrid resin material, MD (Mazic Duro) was fabricated using the same STL files with a milling machine (Roland DG Corporation, Japan).

**Table 2:**
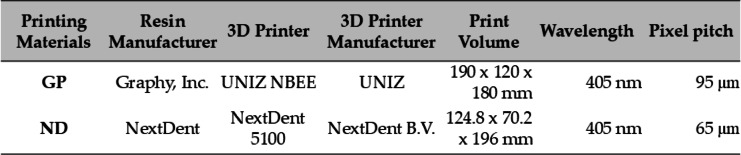
Characteristics of 3D printing devices.

After printing, the samples were ultrasonically cleaned by an ultrasonic cleaner (SH-2240D, SAEHAN Ultrasonic Co., Seoul, South Korea) fully immersed in isopropyl alcohol (99.5%, Samchun Chemical, Seoul, South Korea) for 1 min to remove residual resin on the printed surface. Then, the GP samples were post-cured by using a post-curing unit Tera Harz Cure (THC, Graphy Inc., Seoul, South Korea) at intensity level 2 for a total of 30 min (15 min on both sides) under 95% of Nitrogen^29^. The ND samples were post-cured using a UV post-curing device, LC-3DPrint Box (NextDent, 3D Systems, Rock Hill, SC, USA) for 30 min following the manufacturer’s instructions. The fabricated crown-shaped specimens are shown in Fig. 2a–c.

To comply with ISO 10477 specifications, all 3-point flexural strength test specimens were polished with sandpaper. After, all specimens were ultrasonically cleaned with distilled water. All fabrication steps, including cementation of the specimens, cleaning procedures, and storage conditions, were standardized across all groups. To minimize variability, a single operator performed all procedures following the manufacturers’ instructions.

### Metal die fabrication and crown cementation (Die-Crown unit preparation)

A typodont model of the first molar (KaVo Dental, Biberach/Riß, Germany) was prepared with an axial reduction of 1.5 mm and an occlusal reduction of 1.5–2.0 mm to resemble a full-coverage crown. The model was then scanned using a Medit T310 intraoral scanner (Medit Corp., Seoul, South Korea), and cobalt-chromium (Co-Cr) dies were fabricated through the milling process.

All crowns and dies underwent a trial fitting to ensure passive adaptation. The crowns were cemented onto the dies by a single operator using resin cement (RelyX™ Ultimate, 3 M ESPE, St. Paul, MN, USA) following the manufacturer’s instructions. The specimens were then stored in distilled water at 37 °C for 24 h.

### Fracture test

Each die-crown unit was mounted on a universal testing machine (Instron 3366; Instron Co., Norwood, MA, USA) (Fig. 2e). A stainless steel ball jig with a diameter of 7.5 mm was used to apply force to the occlusal surface. The load was applied at a crosshead speed of 1.0 mm/min until crown fracture occurred. The force required for fracture was recorded in Newtons (N), and fracture occurrence was confirmed through either acoustic detection or mechanical sensing.

### Three-point bending test

The flexural strength and flexural modulus of the tested materials were evaluated using a three-point bending test in accordance with ISO 10477:2019. A universal testing machine (UTM, Zwick Z010; Zwick Poell, Ulm, Germany) was used, with specimens positioned on two supports with a span of 20 mm. A 10 kN load cell applied force at a crosshead speed of 1.0 mm/min until fracture occurred (Fig. 2f).

Flexural strength and flexural modulus were calculated based on the applied load at fracture, specimen dimensions, and displacement under load, following the standardized methodology outlined in ISO 10477:2019 and previous studies^13,17,27,28^.

### Dynamic mechanical analysis (DMA)

To quantify the mechanical dynamics and rheological properties of photocurable 3D-printed resins for provisional restorations, DMA (Q800; TA Instruments, New Castle, DE, USA) was performed over 0.01–100 Hz at 0.1% strain using the dual-cantilever mode. Specimens of two 3D-printed resins (TC-80DP and NextDent C&B) were used (*n* = 1 per resin). The specimen dimensions were 60 × 12.5 × 3.3 mm (TWL)^29^.

### UV rheometer

The rheological properties of the composite resin were evaluated using a rotational rheometer (MCR 702e, Anton Paar, Graz, Austria) equipped with a parallel plate geometry. Measurements were performed under oscillatory mode with a strain amplitude of 1% and a frequency of 1 Hz. The samples were exposed to ultraviolet (UV) light during testing using a UV source (CTLAB ctc4ene4-b63f, 500 W) set at an intensity of 10.3 mW/cm 2 (5% of maximum output). The UV exposure was applied continuously for 250 s while monitoring the resin’s viscoelastic properties.

The UV rheology tests were repeated four times (*n* = 4), and the complex viscosity measurements were performed twice for each group (*n* = 2). The complex viscosity measurement conditions of the composite resin are as follows: the diameter of the disposable parallel plates is 25 mm, the experimental temperature is 25 °C, the plate gap is 100 μm, and the angular velocity is 0.1–100 rad/s^29^.

### Statistical analysis

All statistical analyses were performed using SPSS 25.0 (SPSS Inc., Chicago, IL, USA). The Shapiro-Wilk test was used to assess the normality of the data, while the Levene test was conducted to evaluate homogeneity of variance.

One-way analysis of variance (ANOVA) was performed to compare fracture resistance, flexural strength, and flexural modulus among the three groups: Graphy (GP), Mazic Duro (MD), and NextDent (ND). When ANOVA revealed a significant difference among the groups (α = 0.05), Tukey’s Honest Significant Difference (HSD) test was applied for post-hoc analysis to identify pairwise differences. Mean values and 95% confidence intervals (CIs) were calculated to assess statistical significance.

All statistical tests were conducted using a two-tailed significance level of α = 0.05. The required sample size was calculated using G*Power 3.1.9.7 (effect size = 0.40, α = 0.05, power = 0.80), indicating that at least 10 specimens per group were needed. In this study, 20 specimens per group were tested for flexural strength, and 20 (ND, GP)/19 (MD) specimens for fracture resistance, which exceeded the minimum requirement and ensured sufficient statistical power to detect meaningful differences among groups. To comprehensively evaluate mechanical performance, mean values, standard deviations, and 95% confidence intervals were reported.

## Results

### Fracture resistance

Significant differences were observed in fracture resistance among the tested materials (*p* < 0.001). ND exhibited the highest fracture resistance (4292.35 ± 628.54 N), followed by GP (3340.95 ± 768.21 N, *p* < 0.001) and MD (3028.90 ± 617.70 N, *p* < 0.001). Tukey’s HSD test confirmed that ND had significantly higher fracture resistance than both GP and MD (*p* < 0.001), while no significant difference was found between GP and MD (*p* = 0.324). The results are illustrated in Table 3.

**Table 3:**
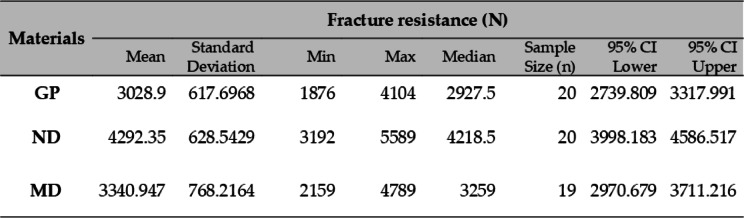
Fracture resistance results analysis.

### Flexural strength and flexural modulus

Significant differences were observed in both flexural strength and flexural modulus among the tested materials (*p* < 0.001 for both properties).

For flexural strength, MD exhibited the highest value (169.99 ± 14.23 MPa), which was about twice as high as ND (75.15 ± 3.76 MPa, *p* < 0.001) and slightly higher than GP (135.62 ± 8.84 MPa, *p* < 0.001). Tukey’s HSD test confirmed that all pairwise comparisons were statistically significant (*p* < 0.001).

For flexural modulus, MD had the highest modulus (15,112.59 ± 284.88 MPa), which was about 6–8 times greater than both GP (2508.50 ± 233.83 MPa, *p* < 0.001) and ND (1837.17 ± 187.95 MPa, *p* < 0.001). Tukey’s HSD test indicated statistically significant differences among all groups (*p* < 0.001).

The results are shown in Table 4.

**Table 4:**
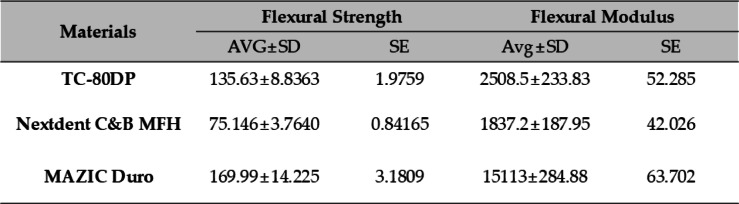
Three-point bending test results (mean ± SD; MPa). AVG,average; SD, standard deviation; SE, standard error.

### DMA (dynamic mechanical analysis)

**Fig. 3:**
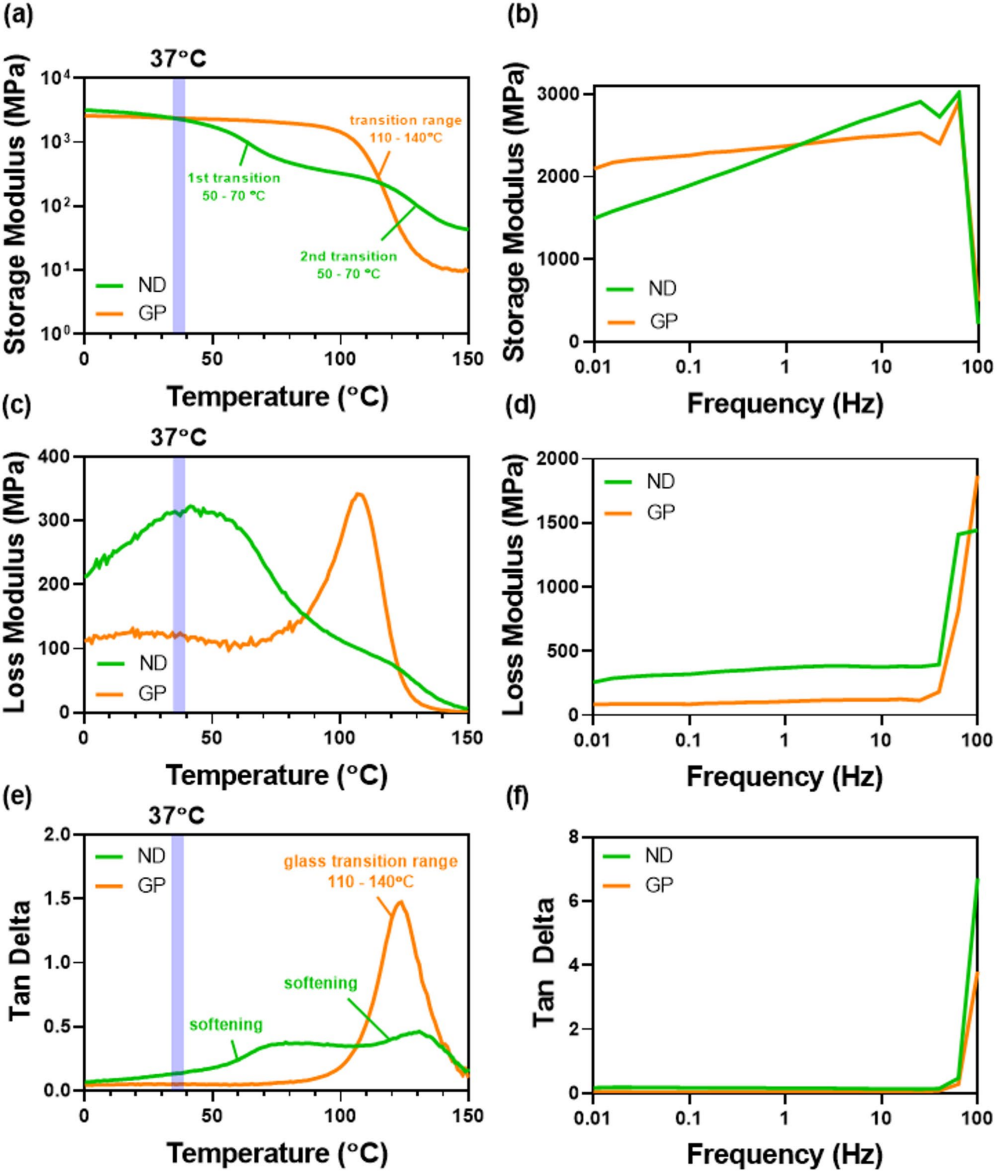
DMA results of 3D-printed restorative materials (NextDent C&B MFH; Graphy TC-80DP A2). (**a**,**b**) Storage modulus (MPa); (**c**,**d**) loss modulus (MPa); and (**e**,**f**) tan δ with respect to (**a**,**c**,**e**) temperature sweep (0–150 °C) and (**b**,**d**,**f**) frequency sweep (0.01–100 Hz) (*n* = 1).

The results from Dynamic Mechanical Analysis (DMA) of the 3D-printed restorative materials, ND and GP, are presented in Fig. 3. The DMA data includes the storage modulus (a, b), loss modulus (c, d), and tan δ (e, f) as functions of temperature (0–150 °C) and frequency (0.01–100 Hz).

In the temperature sweep, storage modulus of both materials exhibits similar storage modulus values at 37 °C, indicating comparable stiffness at body temperature. In Fig. 3a, ND shows fluctuations as temperature increases. In Fig. 3c, GP shows negligible loss modulus at 37 °C, indicating minimal energy dissipation. In contrast, ND demonstrates a slightly higher loss modulus around 300 MPa, suggesting more energy dissipation at body temperature. In Fig. 3e, both materials showed stable behavior up to 37 °C, indicating no significant softening; however, ND began to soften between 50 and 70 °C, suggesting that its structure started to degrade at a lower temperature compared with GP.

In the frequency sweep (Fig. 3b), both materials showed an increase in storage modulus as frequency increases, showing a transition from viscous to elastic behavior. In Fig. 3d, ND exhibited a higher loss modulus than GP, suggesting greater internal friction and energy dissipation. Lastly, in Fig. 3f, tan δ remained at similar values for both materials across the test frequency range, indicating balanced viscoelastic behavior.

### UV rheometer

The UV Rheometer was utilized to analyze the in-situ UV curing characteristics and viscoelastic properties of the materials, specifically focusing on the storage modulus and viscosity.

**Fig. 4:**
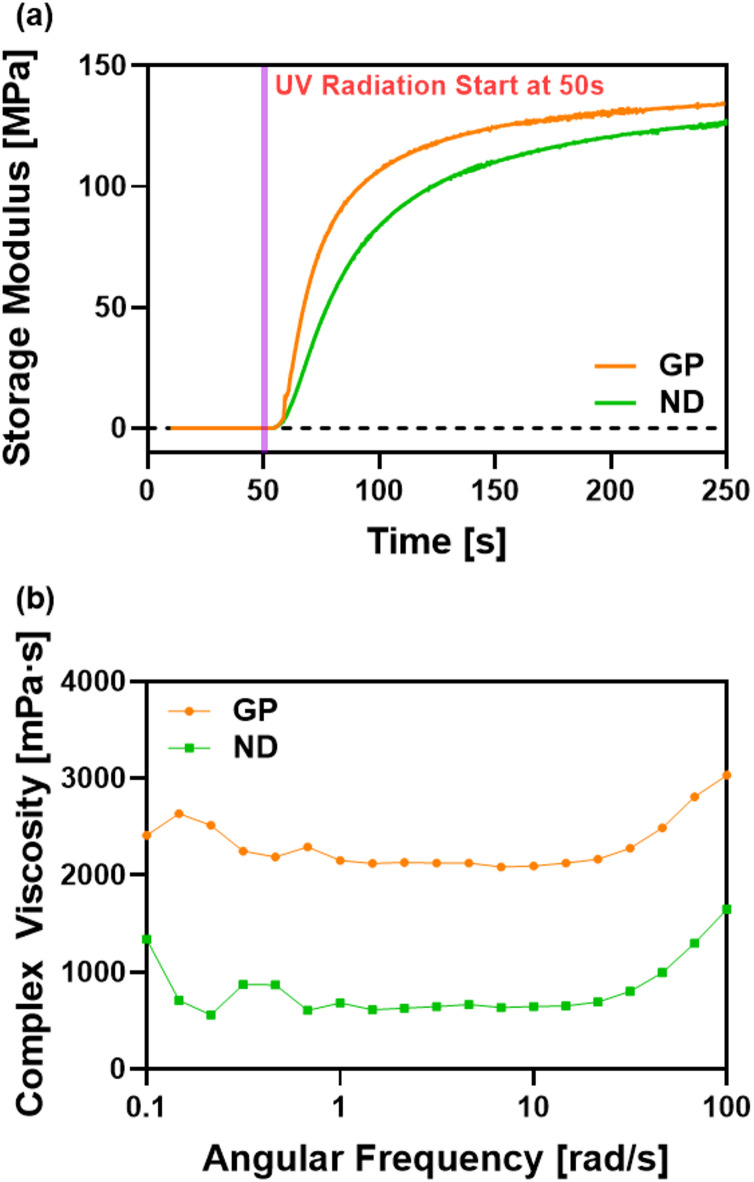
(**a**) Storage modulus (MPa) over time for 3D-printed restorative materials (Graphy TC-80DP A2; NextDent C&B) measured with a UV rheometer; UV irradiation started at 50 s (*n* = 4). (**b**) Complex viscosity (mPa s) of the two materials versus angular frequency (rad s^−1^) (*n* = 2).

The storage modulus values, representing the material’s ability to store energy, were measured for 250 s (~ 4.17 min) and the UV photocurable resins were exposed to UV light at 50 s after the initiation, and was repeated for four times per each test group. As shown in Fig. 4a, after the UV radiation, GP had a steeper slope of increasing storage modulus than ND, and also showed a higher stabilized value of storage modulus than ND. Complex viscosity was also measured with a rheometer across an angular frequency range from 0.1 to 100 rad/s and repeated twice for each test group. In Fig. 4b, GP showed a higher complex viscosity values above 2000 mPa s, whereas ND exhibited a complex viscosity values below 1000 mPa s.

## Discussion

This study evaluated the mechanical properties of two 3D-printed resins (GP and ND) and one milled nano-hybrid resin (MD) for their potential use as long-term provisional materials in pediatric and adolescent patients. These materials were selected because they are clinically available and have been increasingly applied in provisional restorations, including studies involving children and adolescents. In addition, they represent different manufacturing approaches (3D printing vs. milling) and differ in material formulation, such as filler loading and polymer network design, which are known to influence mechanical performance. Previous studies have highlighted critical performance differences between 3D-printed and milled restorative materials under various mechanical and environmental conditions. In particular, print orientation significantly influences the edge strength of definitive 3D-printed resins, with a 90-degree orientation providing superior resistance to marginal chipping compared to milled resins^30^. This reinforces the relevance of assessing structural stability and viscoelasticity in evaluating 3D-printed materials’ durability, particularly under cyclic loads such as bruxism, which is common in growing patients. Among the tested materials, ND showed the highest fracture resistance, whereas MD exhibited the greatest flexural strength and flexural modulus. GP presented intermediate mechanical performance. These differences may be partly attributed to the distinct compositions of each resin. Previous studies have shown that variations in resin formulation, filler content, and polymer matrix significantly influence the mechanical performance of 3D-printed materials, and similar trends have also been reported for CAD/CAM dental blocks^14,17,20,21^. DMA analysis revealed that at 37 °C, both GP and ND had comparable storage moduli. However, ND showed a decline in storage modulus and softening between 50 and 70 °C, while GP maintained stable values up to 100 °C. Under increasing frequencies, both resins exhibited rising storage moduli with stable tan δ values, indicating consistent viscoelastic behavior. GP demonstrated lower loss modulus and more stable mechanical behavior than ND, which exhibited greater internal friction and energy dissipation.

UV rheology results showed that both GP and ND had balanced viscosity and mechanical responses. GP polymerized more rapidly after UV exposure and reached higher storage modulus and complex viscosity (above 2000 mPa s), indicating a denser polymer network. In contrast, ND exhibited slower curing and lower viscosity, forming a more flexible, though potentially less structurally stable, network.

Our results align with previous findings that fracture resistance in 3D-printed and milled materials varies depending on occlusal thickness, and that milled materials typically demonstrate higher flexural strength at increased thicknesses^31^. Although our study used a uniform thickness of 1.5–2.0 mm, ND’s higher fracture resistance suggests that formulation and polymer network density may outweigh thickness as a predictor of flexural strength in some 3D-printed systems. In addition, other studies reported that storage in water or artificial saliva markedly increased nanoindentation creep of 3D-printed composites, with milled resins exhibiting superior dimensional stability^37^. Our viscoelastic results from DMA and UV rheology indirectly support these findings by demonstrating ND’s lower structural stability under thermal and dynamic conditions compared to GP and MD. Incorporating moisture-aging protocols into future testing may better predict intraoral performance. Consistent with earlier research, material-specific differences were also evident. Studies have shown that milled nanohybrid resins exhibit high flexural strength and durability, whereas 3D-printed resins are generally more flexible^8,23,27,28^. MD contains over 80% nanoceramic fillers, contributing to its high strength and flexural resistance, though this composition may also increase brittleness^15,28,32^. Conversely, the optimized polymer matrix of ND enhances its ability to absorb external impact, resulting in superior initial fracture resistance^33,34^.

Although occlusal forces in children and adolescents are generally lower than in adults, factors such as growth, dental arch changes, bruxism, and clenching introduce various stressors^8,9,35–37^. In this study, all tested materials exhibited fracture resistance values far exceeding typical pediatric bite forces (≈ 300–500 N), indicating that they can adequately withstand normal functional loads. Therefore, restorative materials for this group should be evaluated not only for their mechanical strength but also for their viscoelasticity and adaptability, as all of these properties are critical for long-term clinical success. ND’s high fracture resistance and flexibility may be advantageous for short-term protection against sudden occlusal forces or impacts. MD’s superior flexural properties and stability make it more suitable for long-span or high-load provisional restorations, especially in older adolescents. GP, with balanced mechanical and viscoelastic properties, may be preferable in cases involving repeated functional loads. Its stable storage modulus and lower loss modulus under varying temperature and frequency conditions suggest better structural durability under cyclic loading, such as bruxism.

Despite these strengths, each material has limitations. ND may be less suitable for long-term use due to its thermal sensitivity and relatively low flexural strength. GP shows promising viscoelastic stability but may have lower initial strength and be affected by post-curing conditions. MD offers superior strength and thermal stability but may be overly rigid for growing patients and lacks flexibility for chairside modifications.

Therefore, material selection in pediatric and adolescent patients should be individualized based on occlusal force, functional habits, dental development stage, restoration site, and expected duration of use.

This study has limitations. It was conducted in vitro and may not fully replicate intraoral conditions such as moisture, temperature fluctuations, and masticatory forces. Only three specific resin materials were evaluated, limiting generalizability. In addition, the sample sizes were limited to *n* = 1, 4, and 2 for DMA, UV rheology, and complex viscosity testing respectively. Moreover, we did not perform a fractographic failure-mode analysis of fractured specimens, which precluded microscopic confirmation of crack-initiation sites and propagation pathways; SEM-based evaluation would better relate laboratory fractures to clinically observed modes. Long-term fatigue resistance and aging effects were not assessed, and thermocycling, water absorption–desorption measurements, and fatigue testing under simulated intraoral conditions would improve prediction of long-term clinical performance. Investigations using finite element analysis (FEA) to simulate stress distribution under pediatric occlusal loads may provide further insight into clinical outcomes. Moreover, research incorporating microbial colonization and wear resistance assessments could help determine suitability in patients with high caries risk or parafunctional habits such as bruxism. Clinically, randomized controlled trials comparing 3D-printed and milled restorations in children across different age groups and occlusal environments would provide strong evidence for material selection protocols. Additional focus on optimizing post-curing protocols and evaluating new resin formulations tailored for pediatric biomechanics and chairside adjustability would also be beneficial.

These directions will help bridge the gap between laboratory performance and real-world outcomes, ultimately supporting more personalized and durable restorative strategies for young patients.

## Conclusions

This study compared the mechanical performance of two 3D-printed resins and one CAD/CAM-milled nano-hybrid resin for provisional restorations in children and adolescents. ND showed the highest fracture resistance, MD demonstrated superior flexural strength and modulus, and GP exhibited favorable thermal stability and viscoelastic behavior. All materials had fracture resistance values well above typical pediatric bite forces, confirming their adequacy for functional use. Clinically, ND may be advantageous for short-term protection, MD for long-span or high-load cases in older adolescents, and GP for patients with repeated functional stresses. These findings support the use of both milled and 3D-printed resins as viable options, with material selection guided by occlusal load, functional habits, and growth-related factors.

## Data Availability

Data is provided within the manuscript.
